# Dynamic Properties of Heart Fragments from Different Regions and Their Synchronization

**DOI:** 10.3390/bioengineering7030081

**Published:** 2020-07-29

**Authors:** Shin Arai, Kento Lloyd, Tomonori Takahashi, Kazuki Mammoto, Takashi Miyazawa, Kei Tamura, Tomoyuki Kaneko, Kentaro Ishida, Yuuta Moriyama, Toshiyuki Mitsui

**Affiliations:** 1 Department of Physics and Mathematics, College of Science and Engineering, Aoyama Gakuin University, Kanagawa 252-5258, Japan; s-arai@phys.aoyama.ac.jp (S.A.); klloyd@phys.aoyama.ac.jp (K.L.); t-takahashi@phys.aoyama.ac.jp (T.T.); k_mammoto@phys.aoyama.ac.jp (K.M.); tmiyazawa@phys.aoyama.ac.jp (T.M.); k_tamura@phys.aoyama.ac.jp (K.T.); k-ishida@phys.aoyama.ac.jp (K.I.); moriyama@phys.aoyama.ac.jp (Y.M.); 2 Department of Frontier Bioscience, Hosei University, Koganei, Tokyo 184-8584, Japan; tkaneko@hosei.ac.jp

**Keywords:** synchronization, heart tissue fragment, chick embryo

## Abstract

The dynamic properties of the heart differ based on the regions that effectively circulate blood throughout the body with each heartbeat. These properties, including the inter-beat interval (IBI) of autonomous beat activity, are retained even in in vitro tissue fragments. However, details of beat dynamics have not been well analyzed, particularly at the sub-mm scale, although such dynamics of size are important for regenerative medicine and computational studies of the heart. We analyzed the beat dynamics in sub-mm tissue fragments from atria and ventricles of hearts obtained from chick embryos over a period of 40 h. The IBI and contraction speed differed by region and atrial fragments retained their values for a longer time. The major finding of this study is synchronization of these fragment pairs physically attached to each other. The probability of achieving this and the time required differ for regional pairs: atrium–atrium, ventricle–ventricle, or atrium–ventricle. Furthermore, the time required to achieve 1:1 synchronization does not depend on the proximity of initial IBI of paired fragments. Various interesting phenomena, such as 1:n synchronization and a reentrant-like beat sequence, are revealed during synchronization. Finally, our observation of fragment dynamics indicates that mechanical motion itself contributes to the synchronization of atria.

## 1. Introduction

The heart comprises two types of chambers, atria and ventricles, which aid in the circulation of blood in the body through contraction–relaxation cycles in a synchronized manner, also called the heartbeat [1,2]. A normal heartbeat is rhythmically triggered by excitable pacemaker cells and cardiomyocytes. The trigger occurs in the sinoatrial (SA) node located in the right atrium and is followed by the transmission of an electro-impulse, also called action potential, toward the rest of the heart, mainly the ventricular chambers [3,4]. For adequate blood circulation throughout the body, subtle timing delays of the contractions from atrial to ventricular chambers are precisely defined, and the dynamic motion of these chambers is regionally different, which is attributed to the electrical and mechanical properties of the tissues. Once the delicately balanced difference degrades, an electrical or mechanical disturbance is induced, which is recognized as a disease state, i.e., arrhythmia generating an irregular heartbeat. These regional differences are also important for constructing heart tissues from pluripotent stem cells using engineering approaches [5,6].

It is known that the inter-beat interval (IBI) exhibits regional differences as a mechanical property after the heart is separated into blocks, displaying autonomous beat activity in vitro [7,8]. Although there have been intensive investigations into the action potential for estimating IBI using the patch clamp technique for over 50 years, details on mechanical properties have not been well analyzed until recently with the aid of advanced optics and image processing [9,10]. Interestingly, the observed regional differences in mechanical beat motion are not prominent in vitro, as the electrically excitable cardiomyocytes and nonexcitable fibroblasts constituting the heart are different, as are their direction formation activities in vivo [11,12]. In primary cultures on a plastic dish, measurements of contraction amplitude revealed differences between atria and ventricles, but no differences were observed for IBI and contraction speed, likely because the cells were stuck to a hard substrate, which typically slows down IBI [10]. In contrast, dissected heart slices exhibited faster IBI for atria than for ventricles, but the contraction speed measured from edge motions had similar values [9]. Here, we investigated the dynamics of contraction of sub-mm tissue fragments obtained from atria and ventricles on porous filter inserts in 6-well tissue culture plates for over 40 h, using a home-built imaging platform inside an incubator. The fragments in this size range displayed simple rhythmic beats and retained their contractile behavior over tens of hours, which is an indication of vitality [13,14]. We examined the IBI and dynamic properties of atria and ventricles through advanced image processing, such as motion detection and optical flow methods, and discuss the differences here. We also investigated the synchronization of the following tissue fragment pairs at sub–mm scale with regional variations: atrium–atrium (A–A), ventricle–ventricle (V–V), and atrium–ventricle (A–V).

Synchronization processes are a common interest in a wide variety of fields, including biological systems, e.g., circadian heartbeat rhythms and neural networks [1,15]. For the heartbeat, the synchronization of oscillators at various scales from cells to chambers, particularly at sub-mm range, is also of interest for clinical applications, including regenerative medicine and cardiac resynchronization therapy to restore synchrony from arrhythmia [16,17,18,19,20]. In a classical study, the synchronization probability of dissected heart chambers of frogs depended on how close the initial IBI values were for these chambers [7,8]. Prior to complete 1:1 synchronization, partial 1:n synchronization was observed. However, details of the heartbeat dynamics of blocks were not clarified because of limitations in optical instrumentation. As primary cultured cells, the synchronization of cell aggregates on a dish or a pair of sheets has been investigated [21,22,23]. The synchronization of both aggregates and sheets is mostly established within 1 h after they come in contact. However, regional differences in synchronization are discussed, as they both use only ventricular cells. Recently, synchronization of cell aggregates from different age groups of organisms such as mouse and rat have been investigated [24]. Interestingly, an arrhythmia-like behavior that involves blocking action potential propagation was revealed at the cell–cell interface. Here, we present synchronization between paired tissue fragments from atria and ventricles in a sub-mm scale model. Our long time range (>40 h) observation provides regional differences in subtle changes in IBI and beat dynamics toward synchronization. To quantify the degree of synchronization, the Kuramoto model was applied [25,26]. We also evaluated the entrainment time, both optically and electrophysiologically, between paired fragments during the synchronization process. A multi-electrode array (MEA) was used for electrical measurements, with the temporal resolution below the ms range [27]. Toward complete 1:1 synchronization, arrhythmia-like irregular beat sequences, similar to those observed during reentry, were observed on specific regional pairs. Our results on fragment dynamics will likely provide information for mathematical modeling of dynamic activity, including arrhythmia of heart tissue, as the fragment size addresses the gap between the two size scales of individual cells and the whole heart [28,29], where not many experimental observations are available [23,24]. Moving toward the integration of computational studies of the entire heart function on a sub-cellular scale to simulate heart disease and its therapeutic control [30], an understanding of heart dynamics in a tissue–size model with regional differences might also reveal connections between electrical and mechanical properties and help us understand the mechano–electric feedback system [31,32].

## 2. Materials and Methods

### 2.1. Heart Fragments

Heart tissue fragments were obtained from chick embryos after fertilized eggs were incubated at 37 °C for 7 d [14]. All animal experiments were approved by the Life Science Committee of Aoyama Gakuin University. Using an optical microscope, entire hearts were removed from chick embryos using a clean pair of fine scissors, then cut into two pieces, containing atrium and ventricle, followed by further cutting into smaller fragments with a diameter of approximately 0.3 mm by means of microdissection scissors (see Supplementary Figure S1 for details). Fragments 0.3 mm in diameter showed the best beat activity, and the size effect is discussed in Supplementary Figure S1. Typically, one heart yields nearly 15 atrial and 20 ventricular fragments. The heart fragments were transferred onto 0.4 μm pore filter inserts (Falcon Cell Culture Inserts) in 6-well tissue culture plates (Falcon 3046) containing 2 mL culture medium, Dulbecco’s modified Eagle medium/F12 (DMEM:F12), Gluta MAX (TM) supplement (Life Technologies Japan, Tokyo, Japan) containing 10% fetal bovine serum (Nichirei Biosciences Inc., Tokyo, Japan), 100 U/mL penicillin (Meiji Seika Pharma, Tokyo, Japan), and 0.1 mg/mL streptomycin (Meiji Seika Pharma, Tokyo, Japan). Pore filter inserts are an ideal substrate for heart tissue culture because of the efficient media permeation. The pore diameter size of 0.4 μm does not allow for cells to migrate to the other side of the filter surface. In order to make fragment pairs, two fragments originally from the same heart were placed next to each other and slightly pressed to form a physical contact between them. Then, single fragments or pairs of fragments were placed in an array separated by more than 1 mm. The heart fragments on filter inserts were incubated in a humidified atmosphere of 5% CO_2_ at 37 °C. Typically, these fragments show stabilized periodical contraction–relaxation activity within 1 h. For the experiments, 262 fragment pairs from 35 hearts were monitored for the synchronization process.

### 2.2. Monitoring Contractile Activity and Beat Sequences

To evaluate the dynamic properties of beats in tissue fragments and their pairs on filter inserts under culture conditions for over 40 h, we developed a monitoring platform where such beat activity was monitored inside an incubator using an M 500 × DINO USB microscope controlled by LabVIEW software, as shown schematically in Supplementary Figure S2a. For electrophysiological measurements, heart fragments were placed on self-designed multi-electrode array (MEA) chips (Supplementary Figure S2b) which provide the electrical activity for heart fragments. The entrant time of a synchronized fragment pair can be measured by the chips with a time resolution below 1 ms [27]. To analyze beat dynamics and to estimate the inter-beat interval (IBI), a 120 sec movie at 30 Hz was recorded at hourly intervals. The light required for recording inside an incubator was irradiated only during the recording periods. To estimate IBI values, standard 2D image processing from sequential frames of a movie was performed using custom-made codes in MATLAB and LabVIEW (National Instrument Co. Austin, TX, USA).

First, each pixel value of two successive frames is subtracted to recognize the locations of beat motions, containing contractions and relaxations. Second, to identify the timing of beat motion in successive frames, pixel values of a region of interest where beat motion occurs in the subtracted images is plotted vs. time. This generates pulses corresponding to motion timing with a temporal resolution of 33 ms (1/frame rate). When a beat, which indicates contraction followed by relaxation, is slower than 33 ms, double peaks are generated each for contraction and relaxation, such as for a single ventricular fragment, as shown in the lower right panel of Supplementary Figure S3. In contrast, if a beat is faster than 33 ms, it generates a single peak, as seen in a single atrial fragment in the top right panel of Supplementary Figure S3. The time difference between two successive beats is defined as the IBI. The IBI values, presented in this context, were averaged over a 2 min period of a single movie. Practically, the pixel values are integrated over a region of interest where beat motion occurs with the same phase to reduce background noise, and the typical range of integration is 5 × 5 pixels. To draw a velocity field for motion by a beat in a fragment, optical flow analysis was performed using LabView software [33]. Optical flow can track local features in successive frames. Therefore, it enables us to estimate the local velocities of contractions and relaxations of a heart fragment quantitatively. We used the Horn–Schunk algorithm as an optical flow method, which has been previously applied to estimate local velocities of the heart in 3D [34].

## 3. Results

### 3.1. Beat Activity of Single Tissue Fragments of Atria and Ventricles

**Figure 1:**
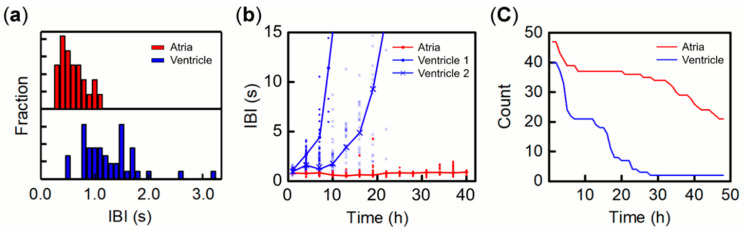
Beat activity of single fragments of atria (red) and ventricles (blue). (**a**) Statistics on inter-beat interval (IBI) of 1 h. (**b**) IBI transitions vs. time for representative fragments of atria (red) and two ventricles (blue). (**c**) Number of single fragments maintaining their beat activity vs. time for atria (red) and ventricles (blue).

After transferring single heart tissue fragments to the surface of porous filter inserts, nearly half of the fragments, 53.3% (8/15) of atrial and 66.7% (16/24) of ventricular fragments, displayed autonomous beat motion with constant rhythm within 1 h under culture conditions (see Video S1: SI movie 1 (atrium) and 2 (ventricle)). The mean inter-beat interval (IBI) over a 2 min time period is summarized in Figure 1a. The atrial IBI values are in the range of 0.3 to 1.1 s, while ventricular IBI values are 0.8 to 2.0 s. The significantly faster IBI for atrial fragments than ventricular fragments is consistent with the regional differences observed in IBI for dissected atria and ventricles from the heart of a day 4.5 chick embryo [9]. In addition, the contraction–relaxation cycle and contraction speed for the atrial fragments are typically below 33 ms and above 0.8 mm/s, respectively. These are also faster than those of the ventricular fragments, approximately 120 ms and 0.5 mm/s, respectively. Details of the beat dynamics of single tissue fragments are presented in Supplementary Figure S3, with local velocity fields overlaid on optical images in a sequence (see also Video S1: SI movie 1 (atrium) and 2 (ventricle)). As displayed in the representative fragments, the contracting direction of atrial fragments (Video S1: SI movie 1) was typically more anisotropic than that of ventricular fragments (Video S1: SI movie 2). Furthermore, atrial fragments showed local beat motion that did not span the entire fragment. In contrast, ventricular fragments showed contraction spread over the entire fragment. Based on our observations of numerous fragments, atrial fragments displayed more anisotropic contractile motion than ventricular fragments. The contractile area for most of the atrial fragments was local, whereas it was rather global for ventricular fragments.

During several hours of incubation, IBI for ventricular fragments become slower and the variability increased with the loss of autonomous beat activity [35,36]. As illustrated by the two blue lines of IBI vs. time for representative ventricular fragments in Figure 1b, these fragments show an IBI > 15 s by 20 h. We define “stop beating” for ventricular fragments as IBI more than 120 s, which is our single monitoring period. Then we count the number of fragments maintaining their beat activity, and the plot of count vs. time is shown in Figure 1c. Almost 50% of ventricular fragments stopped beating by 20 h, and nearly all fragments (38/40) ceased by 30 h. In contrast, a typical atrial IBI is kept nearly constant over tens of hours, and one representative example of this is plotted as red circles in Figure 1b. The IBI variability for atrial fragments was much lower than that of ventricular fragments, as displayed by the distribution of red marks in Figure 1b. However, the amplitude of contraction linearly decreased with time, resulting in an undetectable amplitude for 21 out of 47 atrial fragments by 40 h. Therefore, for atrial fragments, “stop beating” is considered to be contraction amplitude < 5.9 μm, which is below the resolution of our microscope. The other 26 atrial fragments maintained their contraction motion with IBI < 2.0 s during our monitoring period of 40 h. Therefore, the 21 atrial fragments that did not display beat motion might have still exhibited periodic depolarization of action potential, which generates contractions. The contraction amplitude of ventricular fragments also decreased over time, but before the value could become undetectable the beat motion itself ceased completely.

### 3.2. Synchronization of Beats between Paired Fragments from Different Regions

**Figure 2:**
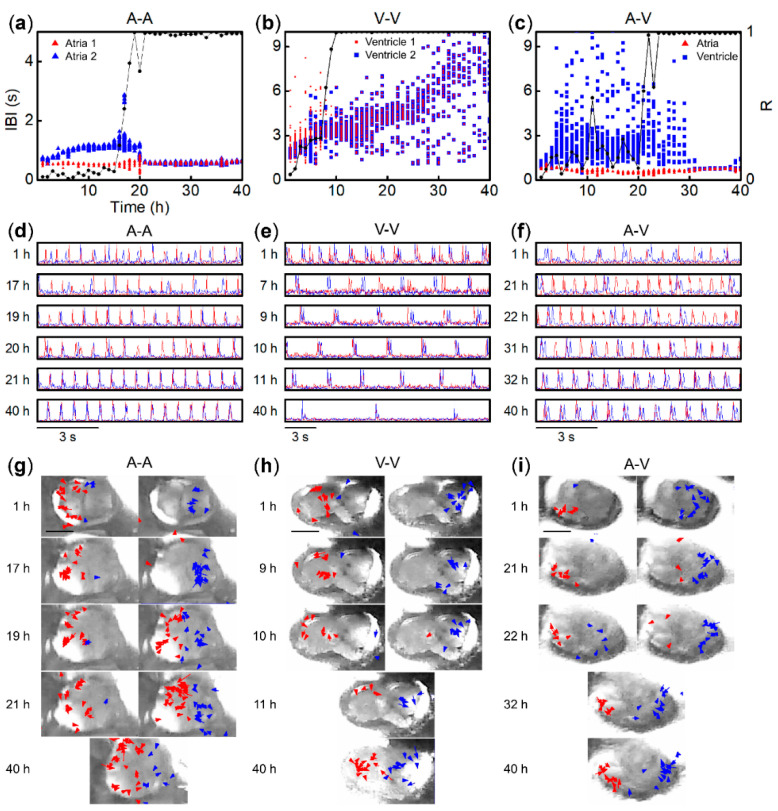
Beat activity of representative tissue fragments of atrium–atrium (A–A), ventricle–ventricle (V–V), and atrium–ventricle (A–V) pairs. (**a**–**c**) Time series of IBI for A–A, V–V, and A–V pairs, respectively. Triangles and squares indicate atrial and ventricular fragments. Red marks indicate fragments showing faster IBI at *t* = 1 h than other fragments, in blue. Line plots exhibit R values estimated by the Karamoto model as an index for synchronization. (**d**–**f**) Beat sequences at selected hours from (a–c), respectively. A beat reveals a single pulse for atrial and double peaks for ventricular fragments. Line coloring follows from (a–c). (**g**–**i**) Contract velocity fields of A–A, V–V, and A–V pairs at selected hours as shown in (a–c), respectively. Overlaid arrows on microscopic images indicate contract velocity with magnitude over 0.4 m/s. Two images at the same hour indicate different contraction timing between fragments. Interfaces between fragments can be recognized at the center, particularly at 1 h time period. Scale bars are 0.3 mm. Contractile motion of these fragment pairs can be visualized in movies (Video S1: SI movie 03–07 for A-A, 08-12 for V-V and A-V for 13-17A-V).

We observed autonomous beat activity of paired tissue fragments with regional variations—atrium–atrium (A–A), ventricle–ventricle (V–V), and atrium–ventricle (A–V)—over 40 h. In Figure 2a–c, scatter plots show the time transition of IBI for A–A, V–V, and A–V, respectively, as representative pairs of each kind. Triangles and squares stand for atria and ventricles, respectively. The red and blue marks represent the initial IBI at *t* = 1 h for fast and slow between the paired fragments. In the first few hours, the IBI transitions over time were similar to the IBI of single fragments shown above. However, a clear transition was observed later, and then each fragment pair achieved 1:1 synchronization, as the red and blue marks show the same IBI after 21 h for A–A (Figure 2a), 9 h for V–V (Figure 2b), and 32 h for A–V (Figure 2c). As shown in Figure 2b, the IBI of V–V fluctuated after 9 h. However, 1:1 synchronization can be recognized by plotting beat timing, displayed as single pulses for atria and double peak pulses for ventricles, as described in detail in the Methods section. Beat timing for selected hours for each pair in Figure 2a–c is shown in Figure 2d–f with the same color scheme. At 1 h, beat timing that appears as red and blue pulses is independent in Figure 2d–f. The beat timing becomes 1:1 synchronized after 21 h for A–A (Figure 2d), 9 h for V–V (Figure 2e), and 32 h for A–V (Figure 2f), as red and blue pulses for beat timing appear nearly simultaneously. Mostly such synchronized beats reveal slight time differences between paired fragments, and the beat order as a pulse sequence indicates their role as pacemakers or followers transferring beats from one to the other. Such time differences, mostly of <0.2 s, between two synchronized beats are considered to be the entrainment time for synchronization. Taken together, all of the results shown in Figure 2 confirm 1:1 synchronization and reveal that synchronized IBI is nearly constant for A–A and A–V, while it broadly fluctuates from 2.0 to 9.0 s for V–V. Regarding pacemaker fragments, the fragments marked in red, beating faster initially, become pacemakers for A–A and A–V (see Figure 2d,f). For A–V, as atrial IBI is faster than ventricular IBI, it is natural for the atrial fragments to become pacemakers. In our statistical investigation discussed in detail below, all atrial fragments became pacemakers for all 23 samples of A–V, showing 1:1 synchronization. In contrast, as seen at 9 h for V–V (Figure 2e), the third synchronized pulse indicates a pacemaker exchange, where a pulse marked in blue arises first. This may be due to the fluctuating IBI of ventricles, resulting in competition for pacemaking between the fragments in a pair. A similar phenomenon was reported, mutual entrainment of rabbit SA nodal cells, where cells reset their beat phases for synchronization [37,38]. This eventually reduces the variability of synchronized beat rates.

Prior to 1:1 synchronization, partial or 1:n synchronization was observed, similar to the previous findings, using paired sliced heart blocks [7,8]. As shown in Figure 2d–f, 1:n synchronization was recognized at 19 h and 20 h for A–A (Figure 2d), 7 h for V–V (Figure 2e), and 22 h and 31 h for A–V (Figure 2f), while 1:2 synchronization was prominent at 19 h for A–A (Figure 2d) and at 31 h for A–V (Figure 2f) a few hours before 1:1 synchronization. The partial synchronization is depicted as digitized marks in the scatter plots of Figure 2a,c. In order to evaluate the process of beat synchronization, including partial synchronization before 1:1, the Kuramoto model was applied to individual beats in paired fragments as coupled phase oscillators [25,26]. The Kuramoto model drives the synchronization parameter (R), representing the level of synchronization from 0 (independent beating) to 1 (completely coupled) including 1:n. The black lines in Figure 2a–c show the R values. The R values prominently increased as early as 16 h for A–A (Figure 2a), 8 h for V–V (Figure 2b), and 21 h for A–V (Figure 2c), where the pulses displayed partial entrainment, as depicted in Figure 2d–f. Then the R value became 1 at 19 h for A–A, 9 h for V–V, and 22 h for A–V, where all beats were entrained. There were notable cases where R dropped from 1 and then regained to 1 at 20 h for A–A (Figure 2a) and 23 h for A–V (Figure 2c). This reduction in the R values is attributed to the IBI fluctuation of a pacemaker, recognized as anomalous pulse sequences at 20 h in Figure 2d. The IBI fluctuations of a pacemaker are similar to a phenomenon called reentry observed in vitro [39] and in vivo [28], which we discuss in detail later. Briefly, the fluctuation starts as a normal pulse sequence from a pacemaker’s red pulse to a follower’s blue pulse, but instantaneously another red pulse arises again, which is considered to be a reentrant. This induces a shorter IBI for this pacemaker.

Next, Figure 2g–i displays contract velocity with magnitude > 0.4 m/s indicated by arrows at selected hours from Figure 2a–c, respectively. These velocity fields were estimated by an optical flow method. The color scheme follows from Figure 2a–c. At 1 h, the contract dynamics, speed, and space distribution resembled those of single fragments described above and in Supplementary Figure S3. The contraction of atrial fragments was faster than that of ventricular fragments (see Video S1: SI movies 1, 6, 11). In contrast, the contraction area was almost spread over the entire fragment for ventricles, while that for atria was local. The contraction area of atria expanded over time, while the speed decreased as expected from the values shown in Figure 2g–i. Another notable change in the velocity fields over time was the contraction locations of follower fragments (blue) before and after synchronization (see also Video S1: SI movie 03-07 for A-A, 08-12 for V-V and 13-17 for A-V). This indicates that the propagation path of the action potential inducing contraction differed before and after synchronization.

### 3.3. Synchronization Probability of Fragment Pairs from Different Regions and IBI Transitions

**Figure 3:**
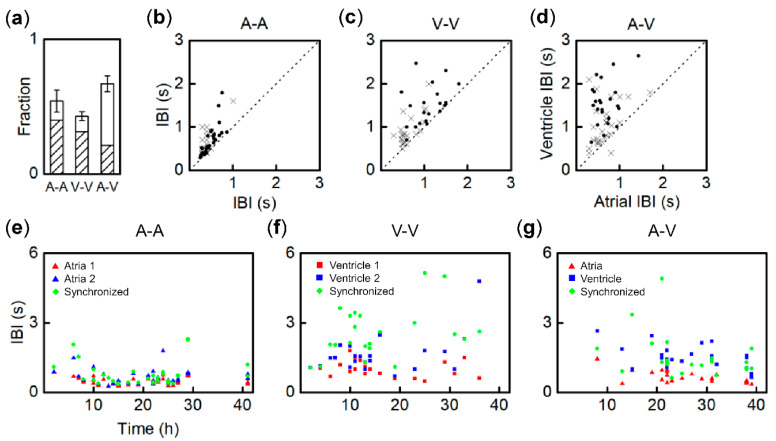
Statistics of synchronization for tissue fragment pairs of A–A, V–V, and A–V. (**a**) Synchronization probability in which the fraction attained 1:1 (diagonal line area). (**b**–**d**) Two IBI values at *t* = 1 h vs. 1:1 synchronization for A–A, V–V, and A–V, respectively. Circles and crosses represent synchronized and nonsynchronized pairs, respectively. For (**b**) and (**c**), faster IBI is plotted as x. For (**d**), atrial IBI is plotted as x. (**e**–**g**) 1:1 synchronization time vs. IBI at *t* = 1 h for A–A, V–V, and A–V (triangle, square), respectively. Synchronized IBI is displayed as green rhombuses. Red marks represent faster IBI at *t* = 1 h, and blue marks represent slower IBI.

In this section, we present a statistical analysis of the synchronization of fragment pairs of three regional kinds. Figure 3a displays the fractions of pairs showing beat entrainment, 54.2% (45/83) for A–A, 42.9% (30/70) for V–V, and 67.0% (73/109) for A–V. The diagonal line area shows the fractions that have 1:1 synchronization: 39.6% (33/83) for A–A, 31.4% (22/70) for V–V, and 21.1% (23/109) for A–V. A–V had the highest probability to entrain, but the lowest to become 1:1 synchronized. A-A had the highest probability to be 1:1 synchronized. Next, we examined initial IBI (*t* = 1 h) of each paired fragment vs. 1:1 synchronization in Figure 3b–d for A–A, V–V, and A–V, respectively. Circles indicate fragments that became 1:1 synchronized by 40 h, while crosses indicate unsynchronized fragments. In Figure 3b,c, the faster IBI at *t* = 1 h is plotted as *x.* In Figure 3d, for A–V, atrial IBI is plotted as *x* because typically atrial IBI is faster than ventricular IBI. Interestingly, there is no correlation between attaining 1:1 synchronization and initial IBI differences between paired fragments, as the circles are not localized near the dotted lines where both IBI values are equal, in Figure 3b–d. In Figure 3c (V–V) and 3d (A–V), the fragment pairs with IBI < 1.0 s show mostly crosses, indicating that the fragments were not able to become 1:1 synchronized. This indicates that ventricular fragments with faster IBI tend to not synchronize with either atria or ventricles. On the other hand, ventricular fragments with slower IBI (>1.0 s) are able to synchronize with atrial fragments, which presumably beat much faster, as shown in Figure 3d. To examine the time to develop 1:1 synchronization vs. initial IBI difference, scatter plots were constructed and are shown in Figure 3e–g for A–A, V–V, and A–V, respectively. The vertical and horizontal axes are for IBI values and time to achieve 1:1 synchronization, respectively. The triangles and squares indicate atria and ventricles, respectively. Red and blue represent faster and slower IBI of paired fragments at *t* = 1 h, respectively, in the same manner as shown in Figure 2. Green rhombuses represent IBI when paired fragments attain 1:1 synchronization. For instance, A–A in Figure 2a shows 0.7 s (red) and 0.9 s (blue) at *t* = 1 h and its synchronized IBI is 0.8 s at *t* = 21 h. These are shown as red and blue triangles and a green rhombus at *t* = 22 h on the horizontal axis of Figure 3e. Statistically, for A–A of Figure 3e, the synchronization time spans mostly from 10 to 30 h regardless of the difference of initial IBI between two fragments. The synchronized IBI (green rhombuses) are typically located between the initial IBI values. On the other hand, the synchronized IBI of V–V in Figure 3f is much slower than the initial IBI, particularly after *t* = 10 h. Among the three pairs, statistically V–V shows the fastest synchronization time, because nearly two-thirds of V–V shows 1:1 synchronization by *t* = 15 h. Interestingly, synchronized V–V fragments (6/22) maintained their beat activity for over 40 h, while nearly all single ventricular fragments lost their beat activity within 30 h (38/40), while 19 out of 23 A–V fragments took longer than 20 h. Similar to A–A shown in Figure 3e, A–V shown in Figure 3g illustrates that the initial IBI difference is not directly correlated with the synchronization time. The synchronized IBI values (green rhombuses) are also located between the initial IBI of atria (triangles) and ventricles (squares). In our observations, atrial fragments became pacemakers for A–V at synchronization. Relative to the synchronized IBI of A–A, that of A–V is slower compared to that shown in Figure 3e,g.

### 3.4. Entrainment Dynamics of Fragment Pairs after 1:1 Synchronization

**Figure 4:**
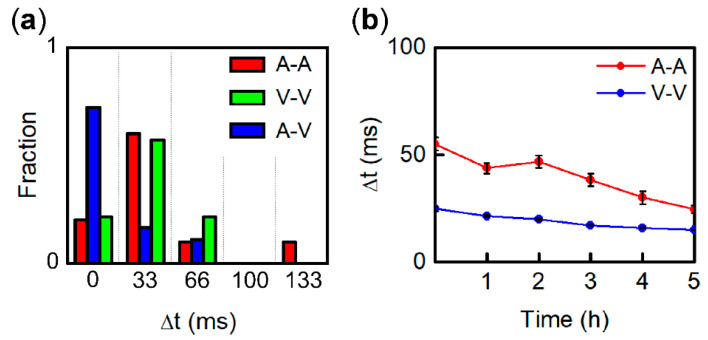
(**a**) Histogram of entrainment time at 1:1 synchronization. (**b**) Entrainment time transition measured by multi-electrode array (MEA) after 1:1 synchronization for A-A and V-V.

Figure 4a shows entrainment time, the slight time difference of synchronized beats in a pacemaker and a follower when they achieve 1:1 synchronization. Although the time resolution is limited by the 30 Hz frame rate of movies, corresponding to 33.3 ms, the differences in entrainment time between V–V and the others is noticeable. It can be seen that 16/22 synchronized V–V fragments beat simultaneously (Δ*t* < 33 ms), while nearly 80% of A–A and A–V beat with a slight time delay of Δ*t* > 33 ms. Entrainment time corresponds to the strength of electrical and mechanical coupling, which decreases over time under culture conditions, mainly reflecting the further development of electrical conduction at the boundary that is often measured between synchronized aggregations of cardiac cells on a Petri dish [23,27]. Figure 4b shows the entrainment time of A–A and V–V measured by using an MEA after 1:1 synchronization is established. Both entrainment times gradually decrease, particularly for A–A as it was halved. This result indicates that conduction across fragments in a paired formation further develops after synchronization is established.

### 3.5. Origin of IBI Fluctuation of Pacemaker

**Figure 5:**
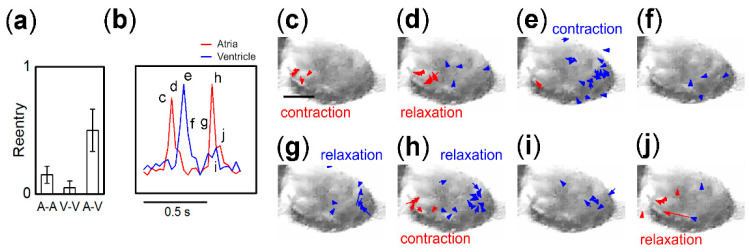
(**a**) Fraction of fragment pairs exhibiting reentry-like beat pulse sequences. (**b**) Example of irregular pulse sequence of A-V pair. (**c**–**j**) Original images of (**b**) with motion indicated by arrows. Contraction (**c**) and relaxation (**d**) occurring in the left fragment, which entrains as a contraction of the right fragment (**e**). While the right fragment shows relaxation from (**g**) to (**h**), the left fragment displays contraction (**h**) followed by relaxation (**j**). Scale bar in (**c**) is 0.3 mm.

Figure 5a displays the fraction of fragment of all pairs generating 1:1 synchronization that showed reentrant-like irregular beat sequences. Such reentrants were evident in nearly half of A–V fragments before 1:1 synchronization was established. As evident from the data presented in Figure 2, irregular beat sequences inducing IBI fluctuation for pacemaker fragments were observed prior to 1:1 synchronization. In particular, at 20 h of A–A (Figure 2f) and 23 h of A–V, shown in Figure 5b, the pacemaker fragment beat twice in a single synchronized beat sequence, similar to observations for the reentry phenomenon [28,39]. The irregular beat sequences of A–V at 23 h are presented in Figure 5b as a representative case, and the contraction–relaxation motion at the time shown in the figure are displayed as microscopic images with local velocity fields in Figure 5c–j. Briefly, the contraction of the atrial fragment, as a pacemaker, appears in Figure 5c, followed by relaxation in Figure 5d. The contraction of the follower ventricular fragment is shown in Figure 5e, followed by relaxation in Figure 5g,h. An irregular beat corresponding to the contraction of the pacemaker emerges simultaneously with the relaxation of the follower in Figure 5h as reverse entrainment occurs (see also Video S1: SI movie 18 of reentry). This irregular beat sequence induces a shorter IBI of 0.33 s for the atrial pacemaker. Such a reentrant beat sequence occurred 16 times out of 227 entrainments (2 min movie at 23 h of A–V). The average of shorter IBI was 0.38 ± 0.09 s (SD), while that of the others was 0.55 ± 0.04 s (SD). A similar time analysis of the other A–V fragment pairs showing reentrants revealed a prominent difference of slow entrainment time from a pacemaker to a follower, corresponding to the Δ*t* between Figure 5c,e. Statistically, when the entrainment time was longer than 0.066 s (framing time 2) for A–V, reentrant-like beat sequences tended to occur. This slow entrainment is likely long enough to enable readiness for another excitation of the atria. With regard to the observation of dynamics, the trigger for the generation of another excitation may be attributed to the physical motion of the ventricle, as electrical signal transmission from the ventricle during relaxation is not plausible because the action potential is in the plateau state.

Reentry, known as a cause of heart arrhythmia, is the generation of a spiral wave of action potential propagation with a contraction motion [28,40]. Two-dimensional (2D) cell sheets with a width of approximately 10 mm × 10 mm or more often display similar spiral waves and are thus an in vitro model for reentry [39,41,42,43]. Such spiral waves are caused by the presence of local inhomogeneity, i.e., tissue or cell aggregate fractures leading to discontinuity or disturbance of the propagation of an action potential. In our experimental model of fragment pairs, an interface between the pair provided local inhomogeneity during the development of electrical and mechanical coupling toward synchronization. The sub-mm width of our fragments rendered them too small to generate such spiral waves. However, we consider that the fragment pairs showing beat reentry, particularly in A–V, could be an in vitro model for artificial arrhythmia, permitting the observation of the process of transformation to a reentrant and recovery from this process at a time scale of hours.

## 4. Discussion

### 4.1. Contractile Function of Single Heart Tissue Fragments

We present the inter-beat interval (IBI) and beat motion of heart tissue fragments at sub-mm scale from atria and ventricles and their pairs on microporous membranes of culture inserts. According to our observations, both singles and pairs of these tissue fragments retained their regional differences. For example, the IBI of atrial fragments was 0.4 s (150 bpm) on average, which was faster than that of ventricular fragments, 1.2 s (50 bpm); both values are similar to the IBI of 190 and 170 bpm for atria and 49 and 82 bpm for ventricles dissected from embryonic chick hearts [9,36]. On a Petri dish, this regional difference in IBI was not observed for primary cells of rats; however, it increased a few times because of the surface stiffness [10,44]. The soft substrate of the membranes of culture inserts aids in persistent spontaneous beat activity of the fragments over a long period of time. Moreover, a higher contraction speed of atria than ventricles can also be recognized on our soft membranes. In contrast, the regional differences in contractile behavior on a Petri dish are due to the primary contraction amplitude between atria and ventricles [10]. These results indicate that microporous membranes are a suitable substrate to investigate the synchronization of fragments with regional differences.

Gradually, slowed down IBI of ventricular fragments and decreased contraction amplitude of atrial fragments over 40 h likely attributed to the outgrowth of fibroblasts, which differentiate into myofibroblasts by sensing the mechanical stiffness of the substrate [45]. Such fibroblasts or myofibroblasts, observed at the periphery of our tissue fragments after 20 h of incubation, are known to make the tissue stiffer and decrease the contraction amplitude and its IBI [46,47]. Because the rhythm of the beat needs to be maintained autonomously in the atria, contraction amplitude is decreased. However, the growth of these fibroblasts or myofibroblasts in the ventricles directly increases the IBI.

### 4.2. Regional Differences in Synchronization Time and Probability

We observed distinct synchronization processes between fragment pairs A–A, V–V, and A–V, particularly the probability and time to attain complete 1:1 synchronization, starting with individual spontaneously beating fragments retaining the regional differences of their contractile behavior. In order to achieve synchronization between cardiac cells and tissues in vitro after their separation followed by reattachment, electrical coupling is required at the attached boundary. Such electrical coupling is mainly attributed to the presence of gap junction channels of connexins [48]. Previously, such reattachment of cell aggregates or layers from sub-mm to mm scale was performed using primary cultured chick embryos or neonatal rat cardiomyocytes on a dish; 1:1 synchronization was completed within an hour [21,22,23]. Because of such a short time scale, gap junction formation from pre-existing precursor proteins was expected. According to our observations of fragment pairs, the time required to establish complete 1:1 synchronization ranged from 8 to 30 h, which was much slower than that required for primary dissociated cells. These findings suggest that the establishment of intercellular mechanical and electrical coupling may be necessary for the attachment of dissected tissue fragments. Cardiac fibroblasts or myofibroblasts, differentiated from fibroblasts, establish such coupling by moving and growing as a process of healing or remodeling after heart injury to regain signal transduction by expressing gap junctions in vivo and in vitro [49,50,51,52]. Because of such a long time scale of 8 to 30 h, these cells may play a significant role in establishing the electrical conduction between the fragments. Furthermore, fibroblasts can contribute to action potential propagation via gap junctions, although the impedance would be high.

Regionally, V–V is rather fast, taking < 15 h for 1:1 synchronization, while A-V is slow, taking >20 h. A–A can take between 10 and 30 h. In vitro, cardiomyocytes from ventricular tissue are mainly used as the primary cells for physiological experiments investigating action potential propagation and synchronization of cell aggregation [49,50]. These cells and their aggregates continuously respond to electrical signals via gap junction proteins, such as Cx43, and transmit the signals further [51]. This indicates that the time scale of synchronization of V-V depends on mechanical and electrical coupling, likely brought about by the growth of fibroblasts with gap junctions at the fragment interface. In contrast, cells from atrial tissues are sensitive to mechanical motion because of the presence of various mechanosensitive channels, which contribute to their autonomous beating activity [36,52]. Particularly during the development of the embryo, atrial cells are rather insensitive to electrical signal transmission, but sensitive to mechanical stretch, causing depolarization [36]. We consider that the reason for the wide range of A–A synchronization time, from 10 to 30 h, could be mechanical motion at the fragment interface. In numerous A–A fragments showing 1:1 synchronization, the time of contractile motion ranged from 1 h until the time synchronization was obtained (other examples of A–A are shown in Supplementary Figure S4). Supplementary Figure S4a shows another example of A–A developing 1:1 synchronization at 27 h. This A-A fragment did not show contractile motion at the fragment interface by *t* = 14 h, and then became 1:1 synchronized at 27 h. Similarly, the A-A fragment pairs with no interface motion took more than 20 h (n = 8/10). In contrast, all A-A fragments (*n* = 11) synchronized by *t* = 15 h showed contractile motion at the fragment interface. This observation indicates that mechanical motion is necessary to develop coupling and synchronization between atrial fragments.

It is interesting that 1:1 synchronization was the slowest (>20 h) for A–V fragments in our experiment. It is natural for atrial fragments to act as pacemakers, since atrial cells have a faster beat rate and a precise rhythm. On the other hand, ventricular cells are less autonomous but tend to synchronize with others, likely via electrical signal propagation through gap junctions. Therefore, the probability of achieving partial synchronization is the highest among the three pairs, as shown in Figure 3a. Naturally, it may take more time to develop electrical and mechanical coupling at the interface between atrial and ventricular cells, which are considered to be different cell kinds [53]. Proliferation of fibroblasts or myofibroblasts originating from either the atria or ventricles may activate signal transmission for beat activity at the interface. It is interesting to note that the 1:1 synchronized IBI of A–V was slower than the IBI of A-A, as depicted with green marks in Figure 3e,g. This is likely related to the action potential duration (APD) keeping action potential high over time. The APD is prominently longer for cardiomyocyte cells from ventricles than atria both in vivo and in vitro [35]. During the APD, the cardiomyocyte cells are inactivated to induce their contraction. Therefore, shortening the APD induces arrhythmia, such as fibrillation, in vivo. For the ventricular fragments, a shortened APD may provide the minimum IBI to contract as 1:1. To make a follower fragment synchronize, the IBI of pacemaker atria must be slower than the minimum ventricular IBI.

Such regional differences in atrial and ventricular fragments imply that a reentrant, such as an anomalous beat sequence, was present in the A–V fragment pairs (Figure 5). The atrial cells with a short APD of 86 ms beat a little more during the APD. Normally, this would occur because of the excitation of one of the atrial cells. However, when an A–V fragment starts to couple electrically and mechanically to achieve synchronization, the entrainment from an atrial fragment to a ventricular fragment, followed by the ventricle’s slow contraction relaxation sequence, results in ventricular relaxation after atrial APD.

As discussed above, atria are vulnerable to mechanical stimulus and generate an excitatory signal per beat, so that the last relaxation motion of the ventricles may trigger another excitation of the atrial fragment [11]. This reentrant-like beat sequence was observed within a few hours of 1:1 synchronization and not thereafter. We suspect that the proliferation of myofibroblasts and gap junction distribution at the interface of fragments could be a part of “remodeling” before a perfect electrical and mechanical coupling is established as myofibroblasts induce arrhythmia [54]. Interestingly, the anomalous beat sequence halted within a few hours in our study. The time scale was too fast to generate further proliferation of fibroblasts or differentiation into myofibroblasts, which could modify the coupling between fragments. Local modifications, including cell orientation and gap junction distribution, may contribute to sTable 1:1 synchronization. In our study, the time duration between an initial beat in a pacemaker atrial fragment and the relaxation motion of the following ventricular fragment in an A–V fragment pair decreased hours after they established 1:1 synchronization as a result of improved mechanical coupling. Nearly all contracting motion of synchronized fragments can be seen to occur simultaneously in 40 Hz movies at 40 h. Therefore, a reentrant-like beat sequence occurs in A-V fragments when the entrain and beat motion are slow, and the ventricle’s relaxation motion triggers the second atrial beat. The electrical coupling at the fragment interface also increases when the latency and entrainment time of synchronization decreases from 200 ms to 1 ms [23].

However, a few questions remain unanswered in our study of fragment synchronization. For example, we observed widening contraction amplitude for both atrial and ventricular fragments once they were 1:1 synchronized. If the proliferation of fibroblasts and myofibroblasts contributes to synchronization, the tissues become mechanically stiffer and contraction amplitudes are likely to decrease in tens of hours, as observed in single fragments [55]. One clear difference between single fragments and pairs of fragments is the existence of an interface. The distribution of fibroblasts and myofibroblasts at the interface needs to be investigated. Further, the distribution of gap junctions, particularly at the interface, should also be examined. We speculate that the regional difference contributes to the distribution of gap junctions. In atria, both Cx40 and Cx43 are activated [48], and while atria rely on Cx40 for fast conductance inducing fast propagation of action potential, ventricles rely on Cx43 with slow conductance. Cx43 enhances the synchronization of primary cultured cardiomyocytes of rat, so a faster synchronization time for V–V may be induced by an increased amount of Cx43 at the V–V interface [16]. To understand how these factors cause fragment synchronization, immunofluorescent staining of the fibroblasts and myofibroblasts or gap junctions, at least Cx40 and Cx43, is necessary for all three pair kinds, compared with and without synchronization. This is our future direction based on the above unanswered questions. As a preliminary result, we observed a higher density of Cx43 at the fragment interface of synchronized A–V. In contrast, myofibroblasts appeared uniformly present at the bottom of the fragments. Immunofluorescent investigations continue to be intensively performed for the three pair kinds, A–A, V–V, and A–V, and then separately imaged with and without synchronization.

Mathematical modelling with electrophysiological measurement is also a strong tool to evaluate the factors of proliferation of fibroblasts and myofibroblasts and/or gap junction redistribution at the fragment interface toward synchronization [56,57]. The changes in electrophysiological consequences, such as action potential duration (APD), are expected from the number of electrical connections between fibroblasts and myocytes [56]. Measuring APD near the fragment interface would indicate the density of fibroblasts/myofibroblasts. In addition, beat rate variability induced by stochastic phenomena such as gating of transmembrane current, releasing Ca2+ via channels, and dynamic behavior of ion channels can be characterized by a mathematical model [57]. Therefore, beat rate variability prior to 1:1 synchronization in our fragment pairs is likely related to such stochastic phenomena originating from gap junctions and ion channels.

To be clinically relevant, the model of heart tissue fragment pairs is oversimplified without the atrioventricular node or His bundle controlling the conduction from atria to ventricles. However, the reentrant-like beat sequence between atrial and ventricular fragments (Figure 5) and the recovery time would be relevant to tachyarrhythmia, as seen in Wolff–Parkinson–White syndrome, because of anomalous coupling between atria and ventricles. Although the size scale is almost 100 times smaller than the heart, it can still be used to quantify the different physical motion of atria and ventricles and their synchronized or arrhythmic behavior, likely to be caused by the proliferation of fibroblasts and myofibroblasts [55] as well as gap junctions in vitro [58]. Heart tissue fragments and pairs at sub-mm scale can be utilized as a model of certain heart diseases. Further, the dynamics of tissue fragments and synchronization coupled with regional differences in the size scale would provide essential information for computational studies of the heart [2]. Recently developed models consider the physical motion of the tissue contributing to electrical activity, presumably by mechanosensitive channels, to simulate the mechano-electric feedback system of the heart [31,32]. However, our fragment model investigates the expected proliferation of fibroblasts and myofibroblasts that remodels the physical properties of the heart and frequently becomes the origin for many diseases [59,60]. Although the distribution of the gap junction and channels remains to be quantified, our study on the dynamics of regionally different fragments and their synchronization process provides information for computational studies and is a potential platform for testing drugs targeted at reentrant tachycardia [61].

## 5. Conclusions

Our investigation on the dynamics of the heartbeat at sub-mm scale with tissue fragment culture inserts in over 40 h of culturing conditions indicates that the dynamic properties differ based on the regional origins of atria and ventricles in the heart and retention of these properties during our observation period, i.e., faster IBI for atria with higher automaticity. Paired fragments display synchronization in beating, and the probability and process of synchronization differ based on the pairs analyzed, A–A, V–V, and A–V. The time required for complete 1:1 synchronization is significantly different based on the pair and does not depend only on the differences between the initial intrinsic IBI for each fragment, but rather on the physical properties of atria and ventricles. Future studies are needed to investigate the origins of synchronization, such as proliferation of fibroblasts/myofibroblasts, ion channels, and gap junction distributions. The reentrant-like beat motion between fragments in a pair when 1:1 synchronization is established could become an in vitro model of arrhythmia because of the similarities in the beat sequence.

## Supplementary Materials

The following are available online at https://www.mdpi.com/2306-5354/7/3/81/s1, Figure S1: (a) Whole heart from E7 chick embryo. (b) Separation of ventricle and atrial fragments. (c) Small fragments of ventricles with diameters of approximately 0.3 mm. (d) A fragment pair on a culture insert. Scale bars are 1 mm in (a)–(c) and 0.1 mm in (d). Figure S2: (a) Schematic figure showing our optical observation system inside an incubator. (b) MEA measurement system. Figure S3: Monitoring beat motions of single fragments of atria and ventricles. Figure S4: A–A fragment pairs with 1:1 synchronization (a) at 27 h and no synchronization (b), Video S1: SI movie 01–18.gif.

## References

[1] Glass L. (2001). Synchronization and rhythmic processes in physiology. Nature.

[2] Nash M.P., Panfilov A.V. (2004). Electromechanical model of excitable tissue to study reentrant cardiac arrhythmias. Progress Biophys. Mol. Biol..

[3] Mangoni M.E., Nargeot J. (2008). Genesis and Regulation of the Heart Automaticity. Physiol. Rev..

[4] Mesirca P., Torrente A.G., Mangoni M.E. (2015). Functional role of voltage gated Ca^2+^ channels in heart automaticity. Front. Physiol..

[5] Hirt Marc N., Hansen A., Eschenhagen T. (2014). Cardiac Tissue Engineering. Circ. Res..

[6] Devalla H.D., Passier R. (2018). Cardiac differentiation of pluripotent stem cells and implications for modeling the heart in health and disease. Sci. Transl. Med..

[7] Marriott Henry J.L. (1956). Atrioventricular Synchronization and Accrochage. Circulation.

[8] Segers M., Lequime J., Denolin H. (1947). Synchronization of auricular and ventricular beats during complete heart block. Am. Heart J..

[9] Sarre A., Maury P., Kucera P., Kappenberger L., Raddatz E. (2006). Arrhythmogenesis in the Developing Heart During Anoxia-Reoxygenation and Hypothermia-Rewarming: An In Vitro Model. J. Cardiovasc. Electrophysiol..

[10] De Souza E.J., Ahmed W., Chan V., Bashir R., Saif T. (2013). Cardiac myocytes’ dynamic contractile behavior differs depending on heart segment. Biotechnol. Bioeng..

[11] Nerbonne J.M., Kass R.S. (2005). Molecular physiology of cardiac repolarization. Physiol. Rev..

[12] Burstein B., Libby E., Calderone A., Nattel S. (2008). Differential Behaviors of Atrial Versus Ventricular Fibroblasts. Circulation.

[13] Easty G.C., Easty D.M. (1963). An Organ Culture System for the Examination of Tumor Invasion. Nature.

[14] Bracke M.E., Parmar V.S., Depass A.L., Stevens C.V., Vanhoecke B.W., Mareel M.M. (2014). Chick heart invasion assay. Methods Mol. Biol..

[15] Strogatz S.H., Stewart I. (1993). Coupled oscillators and biological synchronization. Sci. Am..

[16] Masuda S., Shimizu T., Yamato M., Okano T. (2008). Cell sheet engineering for heart tissue repair. Adv. Drug Deliv. Rev..

[17] Nussinovitch U., Gepstein L. (2015). Optogenetics for in vivo cardiac pacing and resynchronization therapies. Nat. Biotechnol..

[18] Shimizu T., Eberli D. (2011). Myocardial Tissue Engineering. Tissue Engineering for Tissue and Organ Regeneration.

[19] Valentinuzzi M.E. (2017). Why Cardiac Resynchronization Therapy (CRT) is Usually Prescribed along with Automatic Implantable Defibrillation (AID)? Is it a Sensible Decision? Historical Perspective. Int. J. Clin. Cardiol..

[20] Almeida S.O., Skelton R.J., Adigopula S., Ardehali R. (2015). Arrhythmia in Stem Cell Transplantation. Card. Electrophysiol. Clin..

[21] Jongsma H.J., Masson-Pévet M., Tsjernina L. (1987). The development of beat-rate synchronization of rat myocyte pairs in cell culture. Basic Res. Cardiol..

[22] Haraguchi Y., Shimizu T., Yamato M., Kikuchi A., Okano T. (2006). Electrical coupling of cardiomyocyte sheets occurs rapidly via functional gap junction formation. Biomaterials.

[23] Ypey D.L., Clapham D.E., DeHaan R.L. (1979). Development of electrical coupling and action potential synchrony between paired aggregates of embryonic heart cells. J. Membr. Biol..

[24] Agladze N.N., Halaidych O.V., Tsvelaya V.A., Bruegmann T., Kilgus C., Sasse P., Agladze K.I. (2017). Synchronization of excitable cardiac cultures of different origin. Biomater. Sci..

[25] Chen W., Cheng S.C., Avalos E., Drugova O., Osipov G., Lai P.-Y., Chan C.K. (2009). Synchronization in growing heterogeneous media. EPL (Europhy. Lett.).

[26] Kuramoto Y., Nishikawa I. (1987). Statistical macrodynamics of large dynamical systems. Case of a phase transition in oscillator communities. J. Stat. Phys..

[27] Kaneko T., Nomura F., Hamada T., Abe Y., Takamori H., Sakakura T., Takasuna K., Sanbuissho A., Hyllner J., Sartipy P. (2014). On-chip in vitro cell-network pre-clinical cardiac toxicity using spatiotemporal human cardiomyocyte measurement on a chip. Sci. Rep..

[28] Christoph J., Chebbok M., Richter C., Schröder-Schetelig J., Bittihn P., Stein S., Uzelac I., Fenton F.H., Hasenfuß G., Gilmour R.F. (2018). Electromechanical vortex filaments during cardiac fibrillation. Nature.

[29] Walton S., Berger K., Thiyagalingam J., Duffy B., Fang H., Holloway C., Trefethen A.E., Chen M. (2014). Visualizing Cardiovascular Magnetic Resonance (CMR) imagery: Challenges and opportunities. Progress Biophys. Mol. Biol..

[30] Trayanova N.A., Chang K.C. (2016). How computer simulations of the human heart can improve anti-arrhythmia therapy. J. Physiol..

[31] Quarteroni A., Lassila T., Rossi S., Ruiz-Baier R. (2017). Integrated Heart—Coupling multiscale and multiphysics models for the simulation of the cardiac function. Comput. Methods Appl. Mech. Eng..

[32] Nordsletten D.A., Niederer S.A., Nash M.P., Hunter P.J., Smith N.P. (2011). Coupling multi-physics models to cardiac mechanics. Progress Biophys. Mol. Biol..

[33] Hayakawa T., Kunihiro T., Ando T., Kobayashi S., Matsui E., Yada H., Kanda Y., Kurokawa J., Furukawa T. (2014). Image-based evaluation of contraction–relaxation kinetics of human-induced pluripotent stem cell-derived cardiomyocytes: Correlation and complementarity with extracellular electrophysiology. J. Mol. Cell. Cardiol..

[34] Gorce J.-M., Friboulet D., Magnin I.E. (1997). Estimation of three-dimensional cardiac velocity fields: Assessment of a differential method and application to three-dimensional CT data. Med. Image Anal..

[35] DeHaan R.L., Fujii S., Satin J. (1990). Cell Interactions in Cardiac Development. Dev. Growth Differ..

[36] Sabourin J., Robin E., Raddatz E. (2011). A key role of TRPC channels in the regulation of electromechanical activity of the developing heart. Cardiovasc. Res..

[37] Jalife J. (1984). Mutual entrainment and electrical coupling as mechanisms for synchronous firing of rabbit sino-atrial pace-maker cells. J. Physiol..

[38] Verheijck E.E., Wilders R., Joyner R.W., Golod D.A., Kumar R., Jongsma H.J., Bouman L.N., Ginneken A.C.G.V. (1998). Pacemaker Synchronization of Electrically Coupled Rabbit Sinoatrial Node Cells. J. Gen. Physiol..

[39] Hwang S.-M., Kim T.Y., Lee K.J. (2005). Complex-periodic spiral waves in confluent cardiac cell cultures induced by localized inhomogeneities. Proc. Natl. Acad. Sci. USA.

[40] Colli Franzone P., Pavarino L.F., Scacchi S. (2017). Effects of mechanical feedback on the stability of cardiac scroll waves: A bidomain electro-mechanical simulation study. Chaos: Interdiscip. J. Nonlinear Sci..

[41] Borek B., Shajahan T.K., Gabriels J., Hodge A., Glass L., Shrier A. (2012). Pacemaker interactions induce reentrant wave dynamics in engineered cardiac culture. Chaos Interdiscip. J. Nonlinear Sci..

[42] de Diego C., Pai R.K., Dave A.S., Lynch A., Thu M., Chen F., Xie L.-H., Weiss J.N., Valderrábano M. (2008). Spatially discordant alternans in cardiomyocyte monolayers. Am. J. Physiol.-Heart Circ. Physiol..

[43] Sato D., Xie L.-H., Sovari A.A., Tran D.X., Morita N., Xie F., Karagueuzian H., Garfinkel A., Weiss J.N., Qu Z. (2009). Synchronization of chaotic early afterdepolarizations in the genesis of cardiac arrhythmias. Proc. Natl. Acad. Sci..

[44] Engler A.J., Carag-Krieger C., Johnson C.P., Raab M., Tang H.-Y., Speicher D.W., Sanger J.W., Sanger J.M., Discher D.E. (2008). Embryonic cardiomyocytes beat best on a matrix with heart-like elasticity: Scar-like rigidity inhibits beating. J. Cell Sci..

[45] van Putten S., Shafieyan Y., Hinz B. (2016). Mechanical control of cardiac myofibroblasts. J. Mol. Cell. Cardiol..

[46] Thompson Susan A., Copeland Craig R., Reich Daniel H., Tung L. (2011). Mechanical Coupling Between Myofibroblasts and Cardiomyocytes Slows Electric Conduction in Fibrotic Cell Monolayers. Circulation.

[47] Rother J., Richter C., Turco L., Knoch F., Mey I., Luther S., Janshoff A., Bodenschatz E., Tarantola M. (2015). Crosstalk of cardiomyocytes and fibroblasts in co-cultures. Open Biol..

[48] Jansen J.A., van Veen T.A.B., de Bakker J.M.T., van Rijen H.V.M. (2010). Cardiac connexins and impulse propagation. J. Mol. Cell. Cardiol..

[49] Oyamada M., Kimura H., Oyamada Y., Miyamoto A., Ohshika H., Mori M. (1994). The Expression, Phosphorylation, and Localization of Connexin 43 and Gap-Junctional Intercellular Communication during the Establishment of a Synchronized Contraction of Cultured Neonatal Rat Cardiac Myocytes. Exp. Cell Res..

[50] Kojima K., Kaneko T., Yasuda K. (2006). Role of the community effect of cardiomyocyte in the entrainment and reestablishment of stable beating rhythms. Biochem. Biophys. Res. Commun..

[51] Christoffels V.M., Moorman A.F. (2009). Development of the cardiac conduction system: Why are some regions of the heart more arrhythmogenic than others?. Circ. Arrhythm. Electrophysiol..

[52] Zhang H., Shepherd N., Creazzo T.L. (2008). Temperature-sensitive TREK currents contribute to setting the resting membrane potential in embryonic atrial myocytes. J. Physiol..

[53] Fagotto F. (2014). The cellular basis of tissue separation. Development.

[54] Zlochiver S., Muñoz V., Vikstrom K.L., Taffet S.M., Berenfeld O., Jalife J. (2008). Electrotonic Myofibroblast-to-Myocyte Coupling Increases Propensity to Reentrant Arrhythmias in Two-Dimensional Cardiac Monolayers. Biophys. J..

[55] Ongstad E., Kohl P. (2016). Fibroblast–myocyte coupling in the heart: Potential relevance for therapeutic interventions. J. Mol. Cell. Cardiol..

[56] MacCannell K.A., Bazzazi H., Chilton L., Shibukawa Y., Clark R.B., Giles W.R. (2007). A mathematical model of electrotonic interactions between ventricular myocytes and fibroblasts. Biophys. J..

[57] Ponard J.G.C., Kondratyev A.A., Kucera J.P. (2007). Mechanisms of Intrinsic Beating Variability in Cardiac Cell Cultures and Model Pacemaker Networks. Biophys. J..

[58] Jongsma H.J., Wilders R. (2000). Gap junctions in cardiovascular disease. Circ. Res..

[59] Schroer A.K., Merryman W.D. (2015). Mechanobiology of myofibroblast adhesion in fibrotic cardiac disease. J. Cell Sci..

[60] Zeisberg E.M., Kalluri R. (2010). Origins of cardiac fibroblasts. Circ. Res..

[61] Hansen A., Eder A., Bönstrup M., Flato M., Mewe M., Schaaf S., Aksehirlioglu B., Schwörer A., Uebeler J., Eschenhagen T. (2010). Development of a Drug Screening Platform Based on Engineered Heart Tissue. Circ. Res..

