# Supplementary material for: Dynamic Properties of Heart Fragments from Different Regions and Their Synchronization
Source: Bioengineering (Basel). 2020 Jul 29;7(3):81. doi: 10.3390/bioengineering7030081 (PMC7552607; doi:10.3390/bioengineering7030081)
Supplement: Supplementary file 1 [file bioengineering-07-00081-s001.zip › bioengineering-853308-supplemently/bioengineering-853308 suplemently.pdf]

Article

Dynamic Properties of Heart Fragments from Different Regions and Their Synchronization

Shin Arai [1], Kento Lloyd [1], Tomonori Takahashi [1], Kazuki Mammoto [1], Takashi Miyazawa [1], Kei Tamura [1], Tomoyuki Kaneko [2], Kentaro Ishida [1], Yuuta Moriyama [1] and Toshiyuki Mitsui [1,*]

[1] Department of Physics and Mathematics, College of Science and Engineering, Aoyama Gakuin University, Kanagawa 252-5258, Japan; s-arai@phys.aoyama.ac.jp (S.A.); klloyd@phys.aoyama.ac.jp (K.L.); t-takahashi@phys.aoyama.ac.jp (T.T.); k_mammoto@phys.aoyama.ac.jp (K.M.); tmiyazawa@phys.aoyama.ac.jp (T.M.); k_tamura@phys.aoyama.ac.jp (K.T.); k-ishida@phys.aoyama.ac.jp (K.I.); moriyama@phys.aoyama.ac.jp (Y.M.)

[2] Department of Frontier Bioscience, Hosei University, Koganei, Tokyo 184-8584, Japan; tkaneko@hosei.ac.jp

* Correspondence: mitsui@phys.aoyama.ac.jp; Tel.: +81-42-759-6285

Received: 16 June 2020; Accepted: 23 July 2020; Published: date

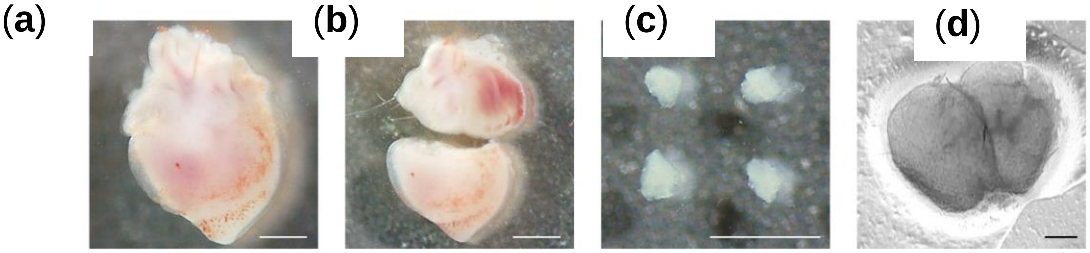

Figure S1. (a) Whole heart from E7 chick embryo**.** (**b**) Separation of ventricle and atrial fragments. (**c**) Small fragments of ventricles with diameters of approximately 0.3 mm. (**d**) A fragment pair on a culture insert. Scale bars are 1 mm in (**a**)-(**c**) and 0.1 mm in (**d**). Because of the highly skilled operation of tissue fragment dissections, the fragments smaller than 0.3 mm of diameter were likely damaged since the probability of generating rhythmic beats was below 25%. The probability raised to 50% by increasing the fragment size, near 0.3 mm of the diameter. Although this probability further increased based on the fragment size, the beat rhythm became irregular, for example, for multi-pacemaking sites in a fragment. In order to investigate synchronization of a pair of tissue fragments, considered as two independent oscillators, we prepared tissue fragments with approximately 0.3 mm diameter. Interestingly, similar sizes of chick heart fragments have been used as host tissues to investigate tumor cell's invasive behavior, termed as Chick Heart Invasion Assay, since the optically visible contractile activity can be considered as a tissue health condition[1,2]. The single tissue fragment gradually increases its size by nearly 10~20% during our monitoring duration of 40 h. From our observations, this is caused by flattening of the fragment shape from spheres to oblate spheroids, caused by the outgrowth of fibroblast cells, likely myofibroblasts, at the periphery of fragments, which firmly adheres to the filter surface of a culture insert[3].

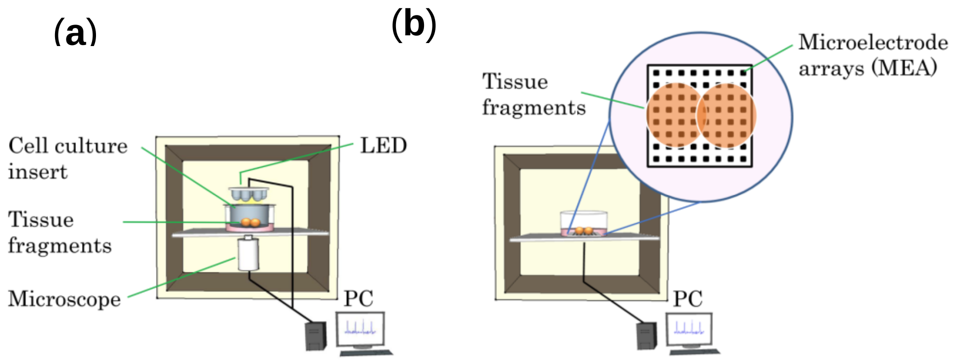

Figure S2. (a) Schematic figure showing our optical observation system inside an incubator**.** (**b**) MEA measurement system.

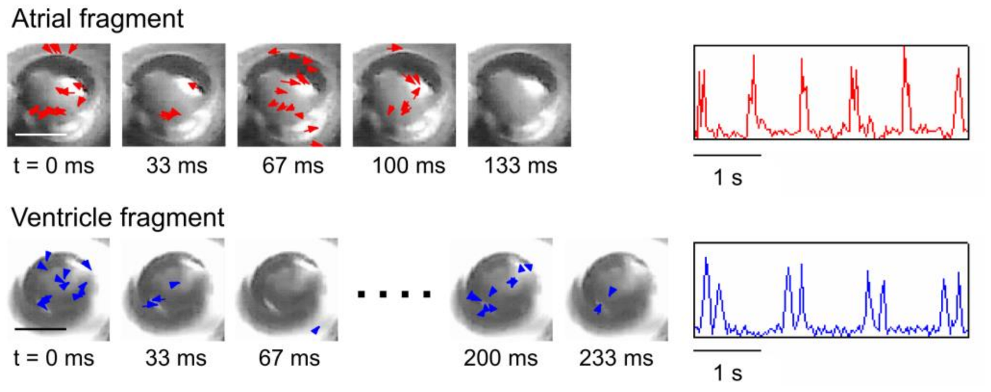

Figure S3. Monitoring beat motions of single fragments of atria and ventricles. Scale bars for microscope images are 0.3 mm. Image frames show individual beat activity. The overlaid arrows, generated by the optical flow method, show local velocities. For atrial fragments, the contraction and relaxation motions show more anisotropy than for ventricle fragments. On the right panel, time traces of the pixel values of subtracted sequential images indicate beat timing for atrium (upper) and ventricle (lower) fragments. A beat motion, contraction followed by relaxation, displays double peaks when the motion is slow, as seen in the case of ventricles. Inter-beat intervals (IBI) can be measured from the time difference between the peaks for sequential beats.

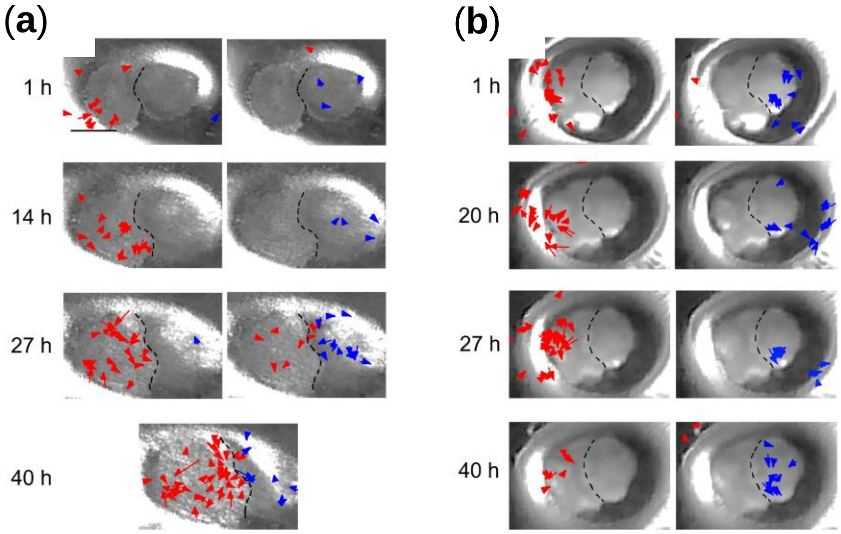

Figure S4 A-A fragment pairs with 1:1 synchronization (**a**) at 27 h and no synchronization (**b**). (**a**) Initially the contractile motion occurs only to the left of the fragment and the fragment interface is static. Then gradually the area of contraction enlarges and the fragment interface, dotted line, starts to contract at 14 h. At 27 h, the fragments become 1:1 synchronized. (**b**) The fragment interface is static from 1 h to 40 h although the area of contraction enlarges slightly in time.

Supplementary movies: SI 1: single atrial fragment; SI 2: single ventricle fragment; SI 3-7 correspond to 1 h, 17 h, 19 h, 21 h, 40h of A-A; SI 8-12 correspond to 1 h, 9 h, 10 h, 11 h, 40 h of V-V; SI 13-17 correspond to 1 h, 21 h, 22 h, 32 h, 40 h for A-V.

1. Easty, G.C.; Easty, D.M. An Organ Culture System for the Examination of Tumor Invasion. Nature 1963, 199, 1104-1105.
2. Bracke, M.E.; Parmar, V.S.; Depass, A.L.; Stevens, C.V.; Vanhoecke, B.W.; Mareel, M.M. Chick heart invasion assay. Methods Mol Biol 2014, 1070, 93-106, doi:10.1007/978-1-4614-8244-4_7.
3. Fahrenbach, J.P.; Mejia-Alvarez, R.; Banach, K. The relevance of non-excitable cells for cardiac pacemaker function. The Journal of Physiology 2007, 585, 565-578, doi:10.1113/jphysiol.2007.144121.
