# Supplementary figures and images for: Dynamic Properties of Heart Fragments from Different Regions and Their Synchronization
Source: Bioengineering (Basel). 2020 Jul 29;7(3):81. doi: 10.3390/bioengineering7030081 (PMC7552607; doi:10.3390/bioengineering7030081)

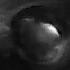

Supplement: Supplementary file 1 [file bioengineering-07-00081-s001.zip › bioengineering-853308-supplemently/bioengineering-853308 Mitsui_Supplementary File/SI movies SI1-18/SI movie 01.gif]

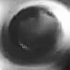

Supplement: Supplementary file 1 [file bioengineering-07-00081-s001.zip › bioengineering-853308-supplemently/bioengineering-853308 Mitsui_Supplementary File/SI movies SI1-18/SI movie 02.gif]

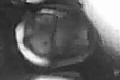

Supplement: Supplementary file 1 [file bioengineering-07-00081-s001.zip › bioengineering-853308-supplemently/bioengineering-853308 Mitsui_Supplementary File/SI movies SI1-18/SI movie 03.gif]

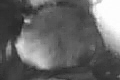

Supplement: Supplementary file 1 [file bioengineering-07-00081-s001.zip › bioengineering-853308-supplemently/bioengineering-853308 Mitsui_Supplementary File/SI movies SI1-18/SI movie 04.gif]

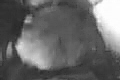

Supplement: Supplementary file 1 [file bioengineering-07-00081-s001.zip › bioengineering-853308-supplemently/bioengineering-853308 Mitsui_Supplementary File/SI movies SI1-18/SI movie 05.gif]

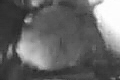

Supplement: Supplementary file 1 [file bioengineering-07-00081-s001.zip › bioengineering-853308-supplemently/bioengineering-853308 Mitsui_Supplementary File/SI movies SI1-18/SI movie 06.gif]

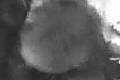

Supplement: Supplementary file 1 [file bioengineering-07-00081-s001.zip › bioengineering-853308-supplemently/bioengineering-853308 Mitsui_Supplementary File/SI movies SI1-18/SI movie 07.gif]

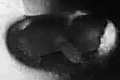

Supplement: Supplementary file 1 [file bioengineering-07-00081-s001.zip › bioengineering-853308-supplemently/bioengineering-853308 Mitsui_Supplementary File/SI movies SI1-18/SI movie 08.gif]

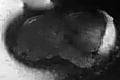

Supplement: Supplementary file 1 [file bioengineering-07-00081-s001.zip › bioengineering-853308-supplemently/bioengineering-853308 Mitsui_Supplementary File/SI movies SI1-18/SI movie 09.gif]

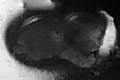

Supplement: Supplementary file 1 [file bioengineering-07-00081-s001.zip › bioengineering-853308-supplemently/bioengineering-853308 Mitsui_Supplementary File/SI movies SI1-18/SI movie 10.gif]

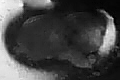

Supplement: Supplementary file 1 [file bioengineering-07-00081-s001.zip › bioengineering-853308-supplemently/bioengineering-853308 Mitsui_Supplementary File/SI movies SI1-18/SI movie 11.gif]

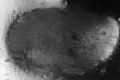

Supplement: Supplementary file 1 [file bioengineering-07-00081-s001.zip › bioengineering-853308-supplemently/bioengineering-853308 Mitsui_Supplementary File/SI movies SI1-18/SI movie 12.gif]

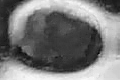

Supplement: Supplementary file 1 [file bioengineering-07-00081-s001.zip › bioengineering-853308-supplemently/bioengineering-853308 Mitsui_Supplementary File/SI movies SI1-18/SI movie 13.gif]

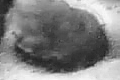

Supplement: Supplementary file 1 [file bioengineering-07-00081-s001.zip › bioengineering-853308-supplemently/bioengineering-853308 Mitsui_Supplementary File/SI movies SI1-18/SI movie 14.gif]

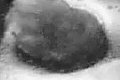

Supplement: Supplementary file 1 [file bioengineering-07-00081-s001.zip › bioengineering-853308-supplemently/bioengineering-853308 Mitsui_Supplementary File/SI movies SI1-18/SI movie 15.gif]

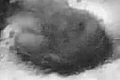

Supplement: Supplementary file 1 [file bioengineering-07-00081-s001.zip › bioengineering-853308-supplemently/bioengineering-853308 Mitsui_Supplementary File/SI movies SI1-18/SI movie 16.gif]

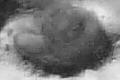

Supplement: Supplementary file 1 [file bioengineering-07-00081-s001.zip › bioengineering-853308-supplemently/bioengineering-853308 Mitsui_Supplementary File/SI movies SI1-18/SI movie 17.gif]

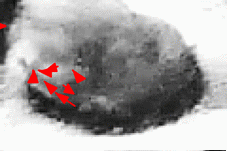

Supplement: Supplementary file 1 [file bioengineering-07-00081-s001.zip › bioengineering-853308-supplemently/bioengineering-853308 Mitsui_Supplementary File/SI movies SI1-18/SI movie 18.gif]
